# Cost-utility analysis of a randomized controlled weight loss trial among lactating overweight/obese women

**DOI:** 10.1186/1471-2458-14-38

**Published:** 2014-01-15

**Authors:** Lars A Hagberg, Hilde K Brekke, Fredrik Bertz, Anna Winkvist

**Affiliations:** 1 Centre for Health Care Science, Örebro University Hospital, Sweden and Örebro University, P.O.Box 1324, SE-701 13 Örebro, Sweden; 2 Department of Internal Medicine and Clinical Nutrition, The Sahlgrenska Academy, University of Gothenburg, P.O Box 459, SE-405 30 Göteborg, Sweden

**Keywords:** Diet, Intervention, Obesity, Lactation, Cost-effectiveness, Sweden

## Abstract

**Background:**

Overweight and obesity among young, adult women are increasing problems in Sweden as in many other countries. The postpartum period may be a good opportunity to improve eating habits and lose weight in a sustainable manner. The aim was to make a cost-utility analysis of a dietary behavior modification treatment alongside usual care, compared to usual care alone, among lactating overweight and obese women.

**Methods:**

This study was a cost-utility analysis based on a randomized controlled and longitudinal clinical diet intervention. Between 2007-2010, 68 women living in Sweden were, after baseline measurement at 8-12 weeks postpartum, randomly assigned to a 12-week dietary behavior modification treatment or control group. Inclusion criteria were: self-reported pre-pregnancy body mass index (BMI) 25-35 kg/m^2^, non-smoker, singleton term delivery, birth weight > 2500 g, intention to breastfeed for 6 mo and no diseases (mother and child). The women in the intervention group received 1.5 hour of individual counseling at study start and 1 hour at follow-up home visits after 6 weeks of intervention, with support through cell phone text messages every two wk. Dietary intervention aimed to reduce dietary intake by 500 kcal/day. The control group received usual care. Weight results have previously been reported. Here we report on analyses carried out during 2012-2013 of cost per quality adjusted life years (QALY), based on the changes in quality of life measured by EQ-5D-3 L and SF-6D. Likelihood of cost-effectiveness was calculated using Net Monetary Benefit method.

**Results:**

Based on conservative assumptions of no remaining effect after 1 year follow-up, the diet intervention was cost-effective. Costs per gained QALY were 8 643 – 9 758 USD. The likelihood for cost-effectiveness, considering a willingness to pay 50 000 USD for a QALY, was 87–93%.

**Conclusions:**

The diet intervention is cost-effective.

**Trial registration:**

ClinicalTrials.gov Identifier: NCT01343238 Registered April 27, 2011.

The regional ethics committee in Gothenburg, Sweden, approved the study on November 15, 2006.

## Background

Overweight and obesity among young, adult women are increasing problems in Sweden as in many other countries [1,2], and pregnancy is associated with persistent weight gain [3-5]. Pre-pregnancy overweight and obesity, and excessive gestational weight gain are risk factors for high postpartum weight retention [6], hence exacerbating a woman’s initial condition with each pregnancy. According to a meta-analysis of long-term weight loss in adults in general, approximately half of the weight loss achieved in successful weight loss programs was regained when the first year after treatment ended [7]. Considering the increased energy requirements of lactation [8,9] as well as new routines and new habits in relation to the caring of the newborn, the postpartum period may be a good opportunity to improve eating habits and lose weight in a more sustainable manner.

We conducted a randomized controlled study, consisting of a 12-week dietary modification program and a 9-month follow-up period, among women who were overweight or obese prior to pregnancy. The study showed significant and clinically relevant results, with a reduction of 2.7 BMI units in the dietary behavior modification treatment group compared to a reduction of 0.3 BMI units in the usual care control group (p < 0,001) [10]. The weight loss in the treatment group was sustained, and even increased, at the 1-year follow-up. The results on body weight and body composition have been reported previously together with detailed descriptions of intervention protocols [10]. However, the cost-effectiveness of the program is unknown.

The aim of this study was to make a cost-utility analysis of a dietary behavior modification treatment, compared to usual care, among lactating overweight and obese women.

## Methods

### Study participants

Between 2007 and 2010, 76 women were recruited to the study from 15 maternity clinics in Gothenburg, Sweden. Inclusion criteria were: self-reported BMI 25-35 kg/m^2^ before pregnancy, non-smoker, singleton term delivery, intention to breastfeed for 6 months, providing less than 20% of the child’s energy intake as a supplementary food, birth weight > 2 500 g, and no known disease in mother or child. Women with mild allergies and stable, medicated hypothyroidism were eligible. The regional ethics committee in Gothenburg, Sweden, approved the study. All participants provided written informed consent.

### Study design

**Figure 1:**
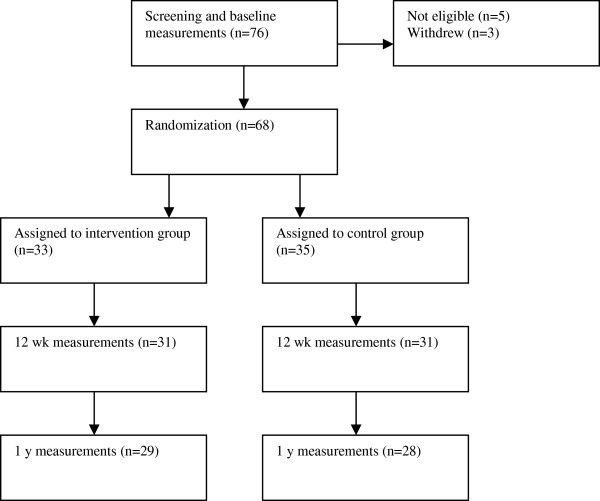
Screening, randomization, and follow-up in the LEVA study.

Baseline measurements were made 8-12 weeks after delivery. Out of the 76 recruited women, 68 women were, after baseline measurement, randomly assigned to either the dietary behavior modification treatment or the control group (Figure 1). Half the participants in each group received physical exercise intervention support consisting of a moderate goal of 8 000 steps per day, within a classical 2×2 factorial design. However, we have previously determined that the physical exercise intervention had no significant effect on daily step-count, total energy expenditure, body weight or body composition at either time point [10]. Hence, in the current cost-effectiveness evaluation we have compared those who received the diet intervention with those who did not receive the diet intervention.

Measurements at baseline, 12 weeks and 1 year are described in detail elsewhere [10]. The women were contacted 6 months after the end of the 12-week intervention and asked about medical conditions and whether they still wanted to participate in the 1-year follow-up. Women who became pregnant during the first 8 months of follow-up were excluded from follow-up measurements. Women who were less than 1 month pregnant at the time of follow-up measurements were kept in the study, because only minimal changes in body weight are likely to have occurred due to pregnancy at that stage [8].

### Intervention

The women in the intervention group received 1.5 hours of individual counseling at the start of the intervention and 1 hour at follow-up home visits after 6 weeks of intervention. Between visits, the women were contacted every 2 weeks via a mobile phone text message with the request to report body weight and encouragement to continue the program.

A dietitian performed the dietary modification counseling to reduce dietary intake by 500 kcal/day (2 092 kJ/day) with a nutrient composition according to the Nordic Nutrition Recommendations [11]. The support to change eating habits consisted of a stepwise plan with practical changes in diet and estimates of their potential effects on weight loss. The plan consisted of four steps: limit sweets, snacks, desserts, sugar-swetened beverages, etc., to 100 g/week, select foods with low fat and sugar content, cover half of their plate with vegetables at lunch and dinner, and reduce portion sizes. The women were provided with a diet plan booklet with a checklist of accomplishment steps for each week, and an electronic body scale for weighing themselves 3 times/week. They were advised to implement one step at a time, and to aim for a rate of 0.5-1.0 kg weight loss per week. Counseling was provided at the research clinic and in follow-up counseling in the women’s homes.

### Control group

Women in the control group received usual care. They were asked not to engage in other lifestyle modification programs during the first 12 weeks of the study.

### Cost effectiveness measurements

Costs were during 2012-2013 calculated based on trial staff’s estimated time consumption per participant. In the calculation, national mean wages [12] and actual expenses of equipment were used. Costs for care center rent and overheads were also estimated. Costs related to the development of the methodology and costs for actitivities entirely related to research methodology were not included in the estimates. Quality of life (QOL) was measured by EQ-5D-3 L in combination with preference scores from a time trade-off measurement in a British population [13,14] as well as by SF-6D (based on SF-36) in combination with preference scores measured with the standard gamble method from a British population [15,16].

All costs were transformed from Swedish currency (SEK) to American dollars (USD) using the exchange rate 1 USD = 7.0 SEK. Costs were recalculated to year 2012 prices using the Swedish consumer price index [17]. All effects and changes in costs were assumed to change linearly between measurement times, and were discounted at 3% per year.

### Health economic analysis method

The analysis in this study was a cost-utility analysis with a societal perspective, using gained quality adjusted life years (QALY) as the measure of health effects [18]. Cost-effectiveness ratios were based on the changes in QOL and costs for the intervention group as compared with the control group. Due to all women being on maternal leave, no loss of productivity was incurred and consequently not included in any analyses. Gained QALY was calculated from the difference in QOL between the intervention and control groups at the follow-up times. Linearly effects were assumed and calculated as follows: For example, if QOL had increased 0.04 more at 3 months and 0.12 more at 1 year in the intervention group than in the control group, the mean change during the first three months would be 0.02 (0.00 + 0.04/2) and during the following nine months 0.08 (0.04 + 0.12/2). Gained QALY for this year would be 0.065 ((0.02×3/12) + (0.08×9/12)).

The timeframe of the analysis is lifelong, but in the base case no effect of the intervention is assumed to remain after 1 year follow-up. The change from baseline to 1 year follow-up seems to have been progressive and is therefore unlikley to suddenly change. Hence, a mean of 1 year remaining effect may be more realistic than no effect after 1-year follow-up. In a sensitivity analysis, two alternative scenarios were calculated; all effects are lost 1 year and 3 years after the follow-up, respectively. Alternative cost effectiveness ratios were also calculated with reduced (50%) effect on QALY and doubled costs of the intervention.

Cost-effectiveness is often calculated from the mean difference in costs and effects, and is calculated between two or more possible treatment options. The use of mean values for cost-effectiveness ratios is associated with uncertainty. In the current analysis, this uncertainty was handled with the Net Monetary Benefit method [19,20]. The method is based on replacing health effects (QALYs), on an individual level, with the amount of money decision makers are willing to pay for a gained QALY. When all data are expressed in money, it is possible to calculate a confidence interval for cost-effectiveness and the likelihood that an intervention is cost-effective in relation to a competing intervention.

A scatter plot of 5 000 bootstrapped incremental cost-effectiveness ratios was created by repeatedly drawing a random sample with replacement using parameters estimated from the study. Individual values were used for gained QALY, and mean values were used for costs related to the intervention (diet and/or physical exercise) that participants received. In such a way, the likelihood that the intervention was cost-effective using several thresholds of willingness to pay for a QALY was calculated. Further, mean Net Monetary Benefit and confidence intervals of Net Monetary Benefit were estimated for these threshold values.

### Statistical method

Data were analyzed according to the intention-to-treat principle, with participants remaining in their original study groups. If data were missing, the last-observation-carried-forward method was used [21-23]. Participants who did not take part in a follow-up were accordingly assumed to have unchanged values. Differences between groups were evaluated using t-test for normally distributed numerical variables. *P*-values <0.05 were regarded as statistically significant differences.

## Results

Of 68 subjects included in the study, 57 completed the 1-year follow-up (Figure 1).

### Costs

Costs were 302.5 USD higher per participant for the women in the intervention group compared to the women in the control group (Table 1).

**Table 1:** Costs (USD) per participant in the LEVA diet weight loss intervention trial

**Type of costs**	**Intervention group**	**Control group**	**Intervention vs. control**
Start of intervention, 1 h 50 min dietitian	61.0	0	61.0
1 home visit, time and travel	74.4	0	74.4
Telephone costs, time and telephone fee	23.0	0	23.0
Participants’ travel expenses	108.9	93.3	15.6
Equipment	78.1	0	78.1
Costs of physical exercise intervention^a^	141.1	141.1	0
Sum of direct costs	486.5	234.4	252.1
Overhead, administration and local costs, 20%	97.3	46.9	50.4
**Sum of total costs per participant**	**583.8**	**281.3**	**302.5**

^a^Including the same kind of costs and the same running time as in the diet intervention, but performed by a physiotherapeut.

### Health effect

After 1 year, the intervention group had increased their QOL in addition to that of the control group with 0.053 (*P* = 0.10) (based on EQ-5D-3 L) and 0.044 (*P* = 0.03) (based on SF-6D) (Table 2). Based on the above data, gained QALY of the intervention group, in addition to that of the control group during the follow-up year, was 0.035 (*P* = 0.19) using the EQ-5D-3 L, and 0.031 (*P* = 0.07) using the SF-6D (Table 3).

**Table 2:** Treatment effect on QOL based on EQ-5D-3 L and SF-6D in the LEVA trial

	**Baseline**		**3 months**		**1 year**	
	**QOL**^**a**^	**P-value**	**QOL change from baseline**	**P-value**	**QOL change from baseline**	**P-value**
**Based on EQ-5D-3 L**						
Intervention group	0.889		+0.013	0.60	+0.022	0.37
Control group	0.880		-0.017	0.56	-0.031	0.15
Difference intervention-control	+0.019	0.48	+0.030	0.43	+0.053	0.10
**Based on SF-6D**						
Intervention group	0.706		+0.048	<0.01	+0.047	<0.01
Control group	0.702		+0.020	0.09	+0.003	0.83
Difference intervention-control	+0.004	0.82	+0.028	0.17	+0.044	0.03

^a^QOL, Quality of Life.

**Table 3:** **Gained QALY for Intervention group beyond that of control group in the LEVA trial**^**a**^

**Assumption of remaining time of effect**	**Based on EQ-5D-3 L**		**Based on SF-6D**	
	**QALY**^**b**^	**P-value**	**QALY**	**P-value**
Follow-up	0.035	0.19	0.031	0.07
Follow-up + 1 year	0.087	0.12	0.074	0.04
Follow-up + 3 years	0.184	0.11	0.156	0.04

^a^Calculated during the 1 year follow-up, and with assumption of remaining effect in 1 year and 3 years. Effects after follow-up years are discounted with 3% per year.

^b^QALY, Quality-adjusted life years.

### Cost-effectiveness

Costs were 8 643 USD (based on EQ-5D-3 L) and 9 758 USD (based on SF-6D) per gained QALY considering the follow-up year. With assumptions of 1 year remaining effect beyond the 1-year follow-up, the cost-effectiveness ratios were 3 477 and 4 088 USD per gained QALY, respectively. With the assumption of a 3-years remaining effect, the ratios were 1 644 and 1 939 USD per gained QALY, respectively.

Net Monetary Benefits were higher for the intervention group than for the control group in all calculations, independent of time of remaining effect, value of a QALY and choice of QOL instrument. Net Moneary Benefts were significantly higher for the intervention group compared to those of the control group using calculations of QALYs based on SF-6D, a willingness to pay for a QALY of at least 100 000 USD, and with an assumption of remaining effect of at least 1 year. With the assumption of 3-year remaining effect, there were significant differences in Net Monetary Benefits between the groups also, with willingness to pay for a QALY set to 50 000 USD (Table 4).

**Table 4:** **Net monetary benefit for Intervention group compared to control group in the LEVA trial**^**a**^

	**QALY**^**b**^ **= 10 000 USD**		**QALY = 50 000 USD**		**QALY = 100 000 USD**	
	**EQ-5D-3 L**	**SF-6D**	**EQ-5D-3 L**	**SF-6D**	**EQ-5D-3 L**	**SF-6D**
Follow-up	46.3	5.4	1441.6	1237.0	3185.7	2776.6
	(-477.4–582.1)	(-324.5–344.0)	(-1137.4–4067.4)	(-398.4–2867.9)	(-1961.7–8426.6)	(-492.2–6030.0)
Follow-up + 1 year	559.9	432.9	4009.7	3374.4	8321.8	7051.3
	(-483.9–1627.1)^c^	(-268.5–1135.6)	(-1180.8–9268.4)	(-106.0–6827.5)	(-2085.7–18833.4)	(100.2–13953.2)
Follow-up + 3 years	1542.6	1251.2	8923.2	7466.1	18148.9	15234.8
	(-609.2–3698.0)	(-173.2–2664.1)	(-1774.5–19682.4)	(367.5-14559.2)	(-3235.4–39619.4)	(1030.6–29422.1)

^a^Using 10 000 USD, 50 000 USD and 100 000 USD as value of a QALY, for the 1 year follow-up and with 1 year and 3 years remaining effect, respectively.

^b^QALY, Quality-adjusted life years.

^c^95% Confidence Interval.

**Figure 2:**
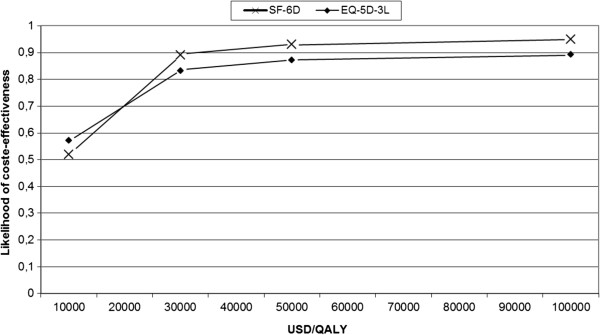
Cost-effectiveness acceptability curve of the base case (the follow-up year).

The likelihood of cost-effectiveness was 0.52 – 0.95 considering follow-up year, when willingness to pay for a QALY was set to 10 000 – 100 000 USD (Figure 2 and Table 5).

**Table 5:** **Probability of cost-effectiveness of the LEVA trial using EQ-5D-3 L and SF-6D to calculate QALY**^**a**^

	**Willingness to pay for a QALY**^**b**^		
	**10 000 USD**	**50 000 USD**	**100 000 USD**
EQ-5D, follow-up	0.57	0.87	0.89
SF-6D, follow-up	0.52	0.93	0.95
EQ-5D, follow-up + 1 year	0.85	0.93	0.94
SF-6D, follow-up + 1 year	0.89	0.97	0.98
EQ-5D, follow-up + 3 years	0.92	0.95	0.95
SF-6D, follow-up + 3 years	0.96	0.98	0.98

^a^Using 10 000, 50 000 and 100 000 USD as value of willingness to pay for a QALY, for the 1 year follow-up and with 1 year and 3 years remaining effect.

^b^QALY, Quality-adjusted life years.

Acceptable cost-effectiveness ratios (around 40 000 USD/QALY) can be obtained also when gained QALY is a fifth of the base case. With halved gained QALY or with doubled cost, the cost-effectiveness ratios are still low (less than 20 000 USD/QALY).

## Discussion

### Principal findings

In this dietary weight loss intervention among overweight and obese lactating women, costs per gained QALY were 8 643 – 9 758 USD based on an assumption of no remaining effect after the follow-up year. With a willingness to pay of 50 000 USD for a QALY, the likelihood for cost-effectiveness was 87 – 93%.

Long-term effects of weight loss programs have generally not been very successful, with half of the weight lost regained again during the first year of follow-up [7]. In contrast, the women in this study actually continued to lose weight between end of the 12-week intervention and the follow-up 9 months later. The reasons for the success of this program are many, including motivation to lose weight after pregnancy, motivation because of parental responsibility, the possibility of implementing new diet routines during maternity leave, and the design/execution of the behavior modification program itself [10]. The perceived increase in quality of life in the diet treatment group may be related to the weight reduction per se, but also to the feeling of being able to control one’s lifestyle and weight, and to make proper choices regarding the diet for themselves and their families.

The approach in this analysis was to calculate the treatment effect as changes in QOL. Additionally, there may be preventive effects against diseases such as type 2 diabetes, heart diseases and osteoarthritis, with possibilities for even better cost-effectiveness ratios. Thus, our results likely underestimate the full cost-effectiveness of the intervention.

Although not all beneficial effects are taken into account, the cost-effectiveness ratio in this study is well below usually accepted cost-effectiveness ratios. There is no formal level of acceptable cost of a QALY in the USA, although 50 000 USD and 100 000 USD are often used [24]. There is also no official level in Great Britain, but The British National Institute for Clinical Excellence applies 20 000 – 30 000 GBP (around 32 000 – 50 000 USD) as acceptable costs, and in Sweden a threshold of 500 000 SEK (37 500 USD) has importance in decisions about subsidized medicine [25].

### Results in relation to other studies

To our knowledge, this is the first analysis of cost-effectiveness of a certain diet intervention trial among overweight or obese women in the postpartum period. However, some analyses of interest have been published in similar fields. In 2011, a model analysis was made estimating cost-effectiveness of weight management interventions following pregnancy [26]. Based on best available data, the preventive effect and the cost-effectiveness were estimated based on changes in weight and BMI after 6 months. Cost per QALY was estimated to 44 144 GBP with a time horizon of 15 years. Consequently, acceptable cost effectiveness ratios can be seen in a short-term treatment perspective as well as in a long-term preventive perspective. Further, cost-effectiveness has been calculated for The Counterweight programme in the UK [27]. This was a family practice-based and theory-based intervention among overweight and obese adults. An economic model study estimated preventive health gains based on changes in BMI after 1 year; the cost-effectiveness ratio was acceptable despite conservative assumptions. Additionally, Roux has shown benefits of weight loss among overweight and obese adults in a lifetime model with the potential of acceptable cost-effectiveness ratio [28]. Dalziel and colleageus have performed model analysis of several nutrition interventions among overweight and obese adults showing acceptable cost-effectiveness ratios of well-performed interventions [29]. Loveman and colleagues in a literature review in 2011 concluded that some evidence exist of cost-effective weight management programs, but caution is required due to analysis methodology [30]. From these data, it is clear that nutrition and weight management interventions among overweight and obese adults have a great potential when it comes to being cost-effective, if the weight loss is sustained. However, all model analyses described above are based on assumptions, and not on empirical data, of future weight development.

### Strengths and weaknesses of the current study

Health economic analyses are often used as a basis for decisions on whether a method should become clinical standard or not. Hence, valid and reliable results are crucial. This requires analyses based on empirical data throughout the follow-up period, as was done in our case. Here, we present results on acceptable cost-effectiveness, which should be sufficient for making decisions on whether the method should be used as clinical standard or not. In addition, a preventive effect can be calculated from measured changes in lifestyle or medical risk markers among participants, in combination with epidemiological data on relationships between changes in these lifestyle factors and risk of diseases or premature death. Hence, the results presented here may underestimate the total expected health gains from the intervention.

Of particular importance for determining the high quality of these analyses is our measurement of QOL. Two instruments were used, EQ-5D-3 L and SF-6D. EQ-5D-3 L is the most frequently used instrument in cost-effectiveness analyses, at least in Europe. However, this instrument may have ceiling-effect problems in such a young and healthy group of participants as ours [31]. SF-6D, based on SF-36, is more recently developed. It is better able to capture QOL in our segment of young and healthy participants, and better able to measure changes because it provides more alternatives to choose from when answering (e.g., 18 000 compared to 243 for EQ-5D-3 L). We think that the validity of our results are enhanced by the fact that the two calculations show similar results.

No measurements were made of participants’ costs for food and time spent cooking. Some studies have shown a healthy diet to be more costly while other studies deem this not to be the case [32,33]. A study from Sweden showed that adhering to a diet similar to the advice in this study is more expensive than adhering to a traditional diet, when differences in energy intake were discounted [33]. However, as the diet led to a lower energy intake there were no significant differences in actual costs between diets. Furthermore, this study took no measurements of health care utilization. However, any such changes may not have been possible to capture with precision due to low health care utilization in general.

The study was, in the original design, performed as a study with four study groups that also included physical activity interventions, both alone and in combination with diet intervention. The physical activity intervention was low targeted with a goal of 8 000 steps a day (which most of the participants reached) in the intervention group as well as in the control group. Hence, the physical activity intervention had no detectable impact on physical activity levels, energy expenditure, body weight or body composition.

Two aspects will have a major impact on the cost-effectiveness: the amount of increase in QOL and how long the increase continues. We think our analyses have handled these aspects as thoroughly as possible. For even more secure results, larger studies with longer follow-up periods are needed. However, to date, our study has the longest follow-up available.

## Conclusion

According to this study, it may be cost-effective to promote weight loss by dietary changes among lactating overweight and obese women. Maternity leave usually entails changes in every day life and this period provides a window of opportunity to implement healthy and sustainable eating habits. Such programs could be offered within ordinary health care.

## Abbreviations

BMI: Body mass index; EQ-5D-3 L: EuroQol 5 dimension questionnaire, 3 levels; GBP: Great Brittian pund; QALY: Quality adjusted life years; QOL: Quality of life; SEK: Swedish krona; SF-6D: Short form 6 dimension questionnaire; SF-36: Short form questionnaire; USD: United States dollar.

## Competing interests

The authors declare no conflicts of interest. The study was supported by grants from the Swedish Research Council (K2009-70X-21091-01-03) and the Swedish Council for Working Life and Social Research (2006-0339). The funders had no role in study design, data collection, analysis, decision to publish or preparation of the paper.

## Authors’ contributions

AW, HB and FB: created the the study concept and design and were responsible for data collection; LH: Outlined the health economic analyzis, analyzed data and wrote the manuscript; AW, HB, FB and LH: interpreted results and critically revised the manuscript. All authors read and approved the final manuscript.

## Pre-publication history

The pre-publication history for this paper can be accessed here:

http://www.biomedcentral.com/1471-2458/14/38/prepub
